# Microbial Community Analysis of Sauerkraut Fermentation Reveals a Stable and Rapidly Established Community

**DOI:** 10.3390/foods7050077

**Published:** 2018-05-12

**Authors:** Michelle A. Zabat, William H. Sano, Jenna I. Wurster, Damien J. Cabral, Peter Belenky

**Affiliations:** Department of Molecular Microbiology and Immunology, Division of Biology and Medicine, Brown University, Providence, RI 02912, USA; michelle_zabat@brown.edu (M.A.Z.); william_sano@brown.edu (W.H.S.); jenna_wurster@brown.edu (J.I.W.); damien_cabral@brown.edu (D.J.C.)

**Keywords:** sauerkraut, microbiome, fermentation, probiotics, high-throughput sequencing

## Abstract

Despite recent interest in microbial communities of fermented foods, there has been little inquiry into the bacterial community dynamics of sauerkraut, one of the world’s oldest and most prevalent fermented foods. In this study, we utilize 16S rRNA amplicon sequencing to profile the microbial community of naturally fermented sauerkraut throughout the fermentation process while also analyzing the bacterial communities of the starting ingredients and the production environment. Our results indicate that the sauerkraut microbiome is rapidly established after fermentation begins and that the community is stable through fermentation and packaging for commercial sale. Our high-throughput analysis is in agreement with previous studies that utilized traditional microbiological assessments but expands the identified taxonomy. Additionally, we find that the microbial communities of the starting ingredients and the production facility environment exhibit low relative abundance of the lactic acid bacteria that dominate fermented sauerkraut.

## 1. Introduction

Sauerkraut, a fermented food made primarily from cabbage, is one of the most well-known varieties of fermented food, dating back to the Roman Empire. Historically, it served as a source of nutrients during the winter months when fresh food was scarce, as proper fermentation preserves the nutritive value of cabbage while creating desirable sensory properties [1,2]. It is most commonly associated with Central and Eastern European cultures, though it can be found in Western European cuisine as well. Sauerkraut is thought to have been part of the American diet since the country’s founding, usually as a cooking ingredient, side dish, or condiment. Its popularity declined beginning in the 1930s as a result of shifting consumer preferences and a lack of product uniformity [1,3]; however, advances in food fermentation science and modern consumer interests have brought sauerkraut renewed popularity in recent years. Today, both mass-produced and artisanal preparations of sauerkraut are widely sold in the United States.

Sauerkraut production and characteristics are largely dependent on the resident microbial community and the fermentation conditions [4]. Though the microbial composition of sauerkraut can vary during the initial stages of fermentation, appropriate fermentation conditions such as temperature and relative ingredient concentration ensure that lactic acid bacteria (LAB) are the dominant microorganisms in the final fermented product. These LAB are of critical importance for successful fermentation; they produce the organic acids, bacteriocins, vitamins, and flavor compounds responsible for many of the characteristic sensory qualities of fermented foods, including extended shelf life, flavor, and nutritional content [5,6,7,8]. Additionally, certain LAB have been purported to act as probiotics that contribute to human health and microbiome stability [9,10]. Though these claims have not yet been fully substantiated by scientists, this perspective has contributed to recent increased consumer popularity and consumption in the United States [11].

Canonical sauerkraut fermentation begins with the initial proliferation of *Leuconostoc mesenteroides*, which rapidly produces carbon dioxide and acid. This quickly lowers the environmental pH, inhibiting the growth of undesirable microorganisms that might cause food spoilage while preserving the color of the cabbage [12]. The action of *L. mesenteroides* changes the fermentation environment so that it favors the succession of other LAB, such as *Lactobacillus brevis* and *Lactobacillus plantarum* [12]. In traditional sauerkraut production, this process proceeds at 18 °C for roughly one month [12]. The combination of metabolites that these organisms produce leads to favorable sensory qualities—the unique flavors, aromas, and textures associated with fermented foods—in the final product [12,13]. The temperature of fermentation also plays an important role in terms of color, flavor, and preservability [12].

Historically, the important species in sauerkraut fermentation were considered to be *L. mesenteroides, L. plantarum, and L. brevis*, which is supported by recent studies [12,14]. In the event of abnormally high heat or salinity, *Enterococcus faecalis* and *Pediococcus cerevisiae* are thought to play a role in the fermentation process [12]. However, these observations were drawn from studies that used culture-based techniques to isolate bacteria, which are inherently biased due to their inability to capture the range of non-culturable bacteria. Recent studies have also identified the genus *Weissella* as important to early fermentative processes [14].

Recent advances in high-throughput sequencing technology have created the potential for highly accurate, culture-independent characterization of the sauerkraut microbiome. The advent of 16S rRNA amplicon sequencing technology has made it possible to systematically analyze the sauerkraut microbiome before, during, and after fermentation. Sauerkraut fermented at warmer temperatures has historically been considered to be of lower quality than sauerkraut fermented at low temperatures; however, current methods of industrial production are turning towards warm-temperature fermentation because it dramatically shortens production time.

Here, we analyze the taxonomic composition of sauerkraut fermented at room temperature over a 14-day fermentation period. Overall, the taxonomic composition of this sauerkraut is in line with the taxonomic composition observed in sauerkraut fermented in the traditional cold temperature range, suggesting that warm-temperature fermentation may be a viable option for producing a sauerkraut with a bacterial community structure that is in line with sauerkraut produced by a more traditional cold-temperature fermentation. This may be of particular interest to industrial and commercial producers, who would be able to speed their production process without sacrificing the taxonomic composition that is at the heart of consumer interest in probiotics and fermented foods.

## 2. Materials and Methods

### 2.1. Sauerkraut Preparation and Sampling Methods

Sauerkraut for this study was sampled from a single 50 lb batch prepared for commercial sale during June 2017 in a facility located near Providence, Rhode Island. Cabbage was salted to a concentration of 2.25% before the addition of caraway seeds (<1% by weight). Ingredient samples were collected in triplicate during a normal production run; 0.5 g of each ingredient were placed into 1.5 mL Eppendorf tubes containing 500 µL of nuclease-free water. The batch of sauerkraut was sealed into airtight plastic drums for the fermentation period. Fermentation was conducted at approximately 21 °C. Successful fermentation was determined by a final pH below 3.6. Fermentation samples were collected in triplicate using Pasteur pipettes from the fermenting sauerkraut at Days 0, 2, 7, 10, and 14. Samples are not true biological replicates, since all triplicates came from the same batch of fermenting sauerkraut; this is a limitation of our study, and future studies should examine the consistency of microbiome dynamics between batches. Packaged, jarred sauerkraut from this producer was purchased from a commercial distributor and processed alongside fermentation samples for microbiome analysis of the finished product.

To sample the production environment, the production table, the industrial sink, and the floor of the production facility were swabbed in triplicate with flocked sterile swabs; these were then stored individually in Zymo Research DNA/RNA Shield Lysis Tubes (Zymo Research, Irvine, CA, USA; Cat: R1103). To sample the air in the facility, empty Petri dishes were left uncovered around the facility throughout the duration of the fermentation period. On Day 14, the Petri dishes were swabbed in the manner described above. After collection, all samples were immediately transported to the laboratory on ice and stored at −80 °C until processing.

### 2.2. DNA Extraction, 16S Library Preparation, and Sequencing

The sauerkraut, environmental, and ingredient samples were processed using the ZymoBIOMICS DNA Microprep Kit (Zymo Research, Irvine, CA, USA; Cat: D4305) according to the manufacturer’s instructions in order to extract DNA. Using the Earth Microbiome Project 16S Illumina Amplicon Protocol, we targeted the V4 hypervariable region of the bacterial 16S rRNA gene using an 806Rb reverse primer (GGACTACCAGGGTATCTAATCC) and a barcoded 515F forward primer (CAGCAGCCGCGGTAAT) [15,16,17,18,19]. PCR amplicons were generated using Phusion High-Fidelity polymerase (New England BioLabs, Ipswich, MA, USA) under the following conditions: 98 °C for 3 minutes, followed by 35 cycles of 98 °C for 45 s, 50 °C for 60 s, and 72 °C for 90 s, and ending with a final elongation at 72 °C for 10 minutes.

PCR amplicon concentrations were analyzed using the Qubit 3.0 Fluorometer and the dsDNA-HS kit (Thermo Fisher Scientific, Waltham, MA, USA) according to the manufacturer’s instructions. Equal amounts of amplicons from each sample were pooled, concentrated, and gel purified using the Machery-Nagel NucleoSpin Gel and PCR Clean-Up kit (Machery-Nagel, Düren, Germany, Cat: 740609) according to the manufacturer’s instructions. The pooled samples were submitted to the Rhode Island Genomics and Sequencing Center at the University of Rhode Island (Kingston, RI, USA) for quality control and sequencing. Amplicons were paired-end sequenced (2 × 250 bp) on an Illumina MiSeq platform using a 500-cycle kit with standard protocols.

### 2.3. Rarefaction and Sequencing Analysis

The raw paired-end FASTQ reads were demultiplexed using idemp (https://github.com/yhwu/idemp/blob/master/idemp.cp) and imported into the Quantitative Insights Into Microbial Ecology 2 program (QIIME2, ver. 2017.9.0, https://qiime2.org/). Raw reads were subsequently deposited into the National Center for Biotechnology Information (NCBI) Sequence Read Archive (SRA) database under the SRA accession SRP145097. The Divisive Amplicon Denoising Algorithm 2 (DADA2) was used to quality filter, trim, de-noise, and merge the data. Chimeric sequences were removed using the consensus method. A feature classifier in QIIME2 trained with the SILVA 99% operational taxonomic unit (OTU) database and trimmed to the V4 region of the 16S was used to assign taxonomy to all ribosomal sequence variants. Contaminating mitochondrial and chloroplast sequences were filtered out of the resulting feature table. The remaining representative sequences were aligned with MAFFT and used for phylogenetic reconstruction in FastTree. Finally, diversity metrics were calculated using the QIIME2 diversity plugin and visualized with Prism (ver. 7.0a, GraphPad, La Jolla, CA, USA).

**Figure 1:**
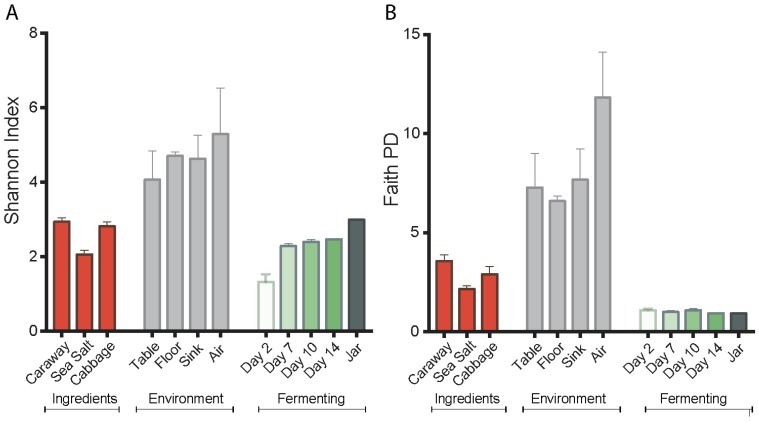
Alpha diversity measures of the sauerkraut, ingredient, and environment samples. (**A**) Shannon index; and (**B**) Faith’s phylogenetic diversity (PD). Error bars represent standard error of the mean.

**Figure 2:**
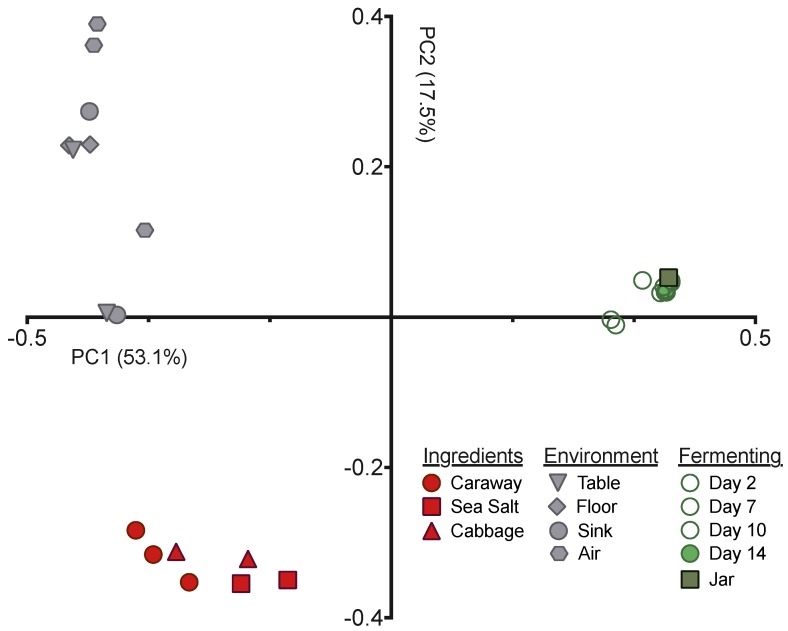
PCoA depicting the unweighted UniFrac distance between fermenting sauerkraut, environment, and ingredient microbiome samples.

**Figure 3:**
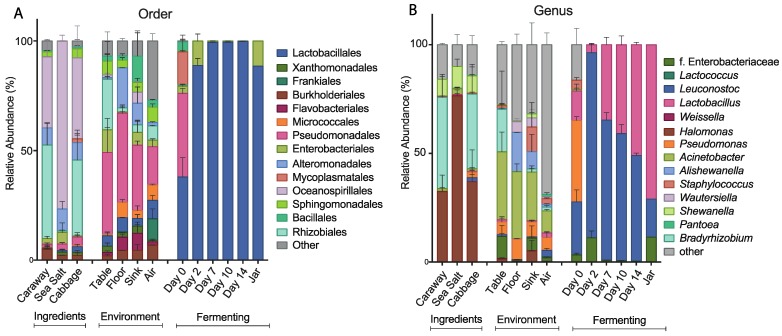
Relative abundance of bacterial taxa in the fermenting sauerkraut, ingredient, and environmental samples at the (**A**) order and (**B**) genus levels. Only the top seven taxa from the fermenting and ingredients/environment sample groupings are shown.

After quality filtering and preprocessing, we determined that 8 of our 37 sequenced samples had fewer than 650 reads, which we deemed insufficient for statistically powerful diversity analysis, and thus a potential source of bias. We therefore removed these read-poor samples from downstream alpha and beta diversity analysis. Five of the discarded samples were distributed, one each, across different ingredient and environmental sample types. Given low variation between the remaining two replicates in these sample types, we feel the two replicates are sufficient for publication. The other three read-poor samples were the triplicate fermenting samples from Day 0. These samples were dominated by contaminating chloroplast reads, which were computationally removed. The remaining bacterial reads were sufficiently low that they presented a problem for alpha and beta diversity measurements. The low abundance of bacterial reads in Day 0 samples and our other samples is likely a reflection of the intrinsically low bacterial abundance of those communities. While this does limit the potential scope of our conclusions, it is an inevitable result of working with low abundance communities. This is reflected by the absence of Day 0 in Figure 1 and Figure 2. To visualize the bacterial community at the Day 0 time point, we used a less restrictive cutoff for sample inclusion in our taxa bar plots—250 reads (Figure 3). This allowed us to recapture all three replicates from Day 0 and gain insight into the structure of these communities in the absence of diversity analysis.

## 3. Results

Alpha diversity values of the environment, ingredients, and fermenting sauerkraut were measured by both the Shannon diversity index and Faith’s phylogenetic diversity. These values reveal a reduction in bacterial diversity for fermenting sauerkraut as compared to the starting ingredients and environment (Figure 1). However, we observ­e contrasting alpha diversity patterns between the Shannon diversity and the Faith’s phylogenetic diversity metrics during the fermentation process. The Shannon diversity index indicates a successive increase in alpha diversity of sauerkraut over time while Faith’s phylogenetic diversity suggests a constant low alpha diversity. We attribute this to the fact that the Shannon diversity index segments closely related and possibly overlapping LAB into separate taxa. This generates a false appearance of diversity. By contrast, Faith’s phylogenetic diversity index uses branch lengths as the basis for assigning diversity metrics, and does not separate LAB with the same level of granularity. Nevertheless, the low level of diversity in the fermenting product shown by both plots is likely the result of selective pressures in the fermentation environment, including low pH, anaerobic conditions, and high salinity. This indicates successful fermentation.

Next we employed principal coordinates analysis (PCoA), with the unweighted UniFrac distance, to visualize the differences in community structures between our samples (Figure 2). The samples exhibited clear clustering by sample type—raw ingredient, environment, or fermentation time point—indicating that the bacterial communities present have significant variation from one another. This is expected for the environmental samples versus raw ingredients or fermentation time points, but was surprising for the raw ingredients versus the fermenting sauerkraut. The Day 0 sample derived from the initial ingredient mixture is much more similar to the Day 14 sauerkraut community than it is to the raw ingredients (Figure 2). This suggests that the selective pressures intrinsic to fermentation have strong and immediate impacts on the bacteria found on and in the raw ingredients.

To further characterize the bacterial community found in each of the collected samples, we examined the taxonomic structures of our bacterial communities at the order and genus levels (Figure 3). At the order level, we see differences in bacterial composition between the raw ingredient samples, the environmental samples, and the fermentation samples (Figure 3A). The raw ingredient and environmental site categorizations are fairly similar to each other. Sea salt differs slightly from the cabbage and caraway seed, which may be because salt is not a plant product and it likely presents a strong halophilic selection pressure. Overall, these two categories of samples are markedly different from the bacterial communities found during fermentation. The Day 0 sample contains significantly more bacterial taxa than the subsequent time point samples, and illustrates a precipitous drop in the number of bacterial species present over the first 48 hours of fermentation. The most abundant bacterial order in the Day 0 fermentation sample is Pseudomonadales, which is also a high abundance order in all of the environmental samples. This suggests that the environment plays some role in establishing the initial bacterial community of the combined ingredients. After two days, the most abundant bacteria present are of the Lactobacillales order, which is expected in the case of successful fermentation. This pattern persists throughout fermentation and jarring.

At the genus level, we find many of the same trends hold (Figure 3B). The three ingredient samples look the most similar at this level, with *Halomonas* common to and prevalent in all three of the samples. The environmental samples continue to show similarity, and the lack of similarity between the environmental samples and the Day 0 fermentation samples persists here as it did at the order level. LAB dominate the other fermentation samples as they did at the order level, with *Leuconostoc* and *Lactobacillus* as the dominant genera. This is in line with the results published by Pederson and Albury 1969, which showed *Leuconostoc* and *Lactobacillus* as main players in the sauerkraut fermentation process [12].

## 4. Discussion

Overall, our results show that, despite the warmer and more rapid temperature fermentation process used to produce the sauerkraut analyzed here, the bacterial community is in line with that of more traditional, colder fermented cabbage products. Over the first 48 hours of fermentation, the microbial community of sauerkraut experienced a precipitous drop in the number of bacterial taxa present, likely due to the strong selective pressures of high salinity and acidity in the fermentation environment. Over the remainder of the fermentation period, LAB remained the dominant organisms present in the community. Both patterns are indicative of successful fermentation.

Perhaps more surprising were the relationships between the microbial communities of the starting ingredients, the fermentation environment, and the fermenting sauerkraut. The major LAB found in fermenting sauerkraut were present only in extremely low levels in the starting ingredients, which may suggest that only trace amounts of LAB are necessary to initiate fermentation. It is also possible that the abundance of fermentative sauerkraut LAB found around the production facility—especially in the air—might contribute to the inception of the fermentative community, acting as a starter culture. The presence of LAB in the environment may also be a direct result of sauerkraut being fermented within it. These hypotheses require further investigation.

Previous studies have used culture- and sequencing-based methods to elucidate the fermentative microbial community of sauerkraut. Culture-based methods have shown that the major LAB involved in sauerkraut fermentation are *E. faecalis*, *L. mesenteroides*, *L. brevis*, *P. cerevisiae*, and *L. plantarum;* while sequencing-based methods highlight the *Lactobacillus* and *Leuconostoc* species in addition to *Weissella* [11,13]. Our results using 16S rRNA sequencing paralleled these expectations and expanded on previous knowledge, identifying *Leuconostoc, Lactobacillus*, and Enterobacteriaceae in addition to a variety of LAB not previously detected, such as *Lactococcus*.

Our results are also in line with the canonical microbial communities of other fermented vegetable foods. Xiong et al. found that *Lactobacillus* and *Leuconostoc* species were the primary bacteria in the fermentation of Chinese sauerkraut, *pàocài* [20]. Numerous studies have shown that the kimchi bacterial community is dominated by *Weisella, Lactobacillus,* and *Leuconostoc* species [21,22,23]. A study of traditional Vietnamese fermented vegetables, such as mustard and beet ferment (*dua muoi*) and fermented eggplant (*cà muối*), found a predominance of *Lactobacillus* species in fermentation [24].

Our results suggest that warmer and more rapid production can yield fermented sauerkraut with a similar microbial community to sauerkraut produced by traditional fermentation methods. This may mean that a quick-fermented process is a viable option for industrial production of fermented cabbage foods. This may be of interest to commercial producers, as it would allow them to speed and scale-up production without sacrificing the integrity of the fermentative bacterial community, which is central to the purported probiotic benefits of sauerkraut and other fermented foods.

While the analyzed communities were roughly similar to previously published sauerkraut data, we cannot yet claim that the products are identical or that the production processes are interchangeable. There are multiple metrics—physical, sensory, and nutritive—that were not investigated as part of this study and could possibly vary between the two types of sauerkraut. We anticipate that diminished appearance, shelf life, taste, and nutritive value of warmer fermented sauerkraut could negatively impact its commercial viability. Therefore, additional studies and measurements of these qualities are required before widespread commercial implementation of this fermentation technique.
